# The effects of varicocelectomy on the DNA fragmentation index and other sperm parameters: a meta-analysis

**DOI:** 10.1186/s12610-020-00112-6

**Published:** 2020-09-10

**Authors:** Ponco Birowo, J. Rahendra Wijaya, Widi Atmoko, Nur Rasyid

**Affiliations:** Department of Urology, Faculty of Medicine Universitas Indonesia, Cipto Mangunkusumo Hospital, Pangeran Diponegoro Street No. 71, Senen Subdistrict, Central Jakarta District, Jakarta, 10430 Indonesia

**Keywords:** Varicocele, Varicocelectomy, DNA fragmentation, Sperm analysis

## Abstract

**Background:**

Varicocele is one of the most common causes of reversible male infertility, and 15% of the varicocele patients with normal semen analysis are diagnosed as infertile. According to the current guidelines, varicocelectomy is indicated based on abnormal sperm parameters and not abnormal DNA fragmentation index (DFI) values. Thus, in this study, we performed a meta-analysis of the effects of varicocelectomy on the DFI and other conventional sperm parameters, and determined whether DFI could be used to indicate varicocelectomy for varicocele patients.

**Results:**

Through an electronic search of the PubMed, Scopus, EBSCO, and Cochrane databases, we included 7 prospective studies including a total of 289 patients in this meta-analysis. The results showed that varicocelectomy significantly reduced DNA fragmentation (mean difference: − 6.86; 95% confidence interval [CI]: − 10.04, − 3.69; *p* < 0.00001) and improved sperm concentration (mean difference: 9.59; 95% CI: 7.80, 11.38; p < 0.00001), progressive motility (mean difference: 8.66; 95% CI: 6.96, 10.36; p < 0.00001), and morphology (mean difference: 2.73; 95% CI: 0,65, 4.80; *p* = 0.01).

**Conclusion:**

Varicocelectomy reduced DNA fragmentation and improved sperm concentration, progressive motility, and morphology. Additionally, the analysis showed that an abnormal DFI measurement should be considered as an indication for varicocelectomy.

## Background

Varicocele is often associated with infertility and reduction in sperm quality. It is one of the most common causes of reversible male infertility. The prevalence of clinically relevant varicocele ranges from 5 to 20%. It has affected 19–41% of men with primary infertility and 45–81% of men with secondary infertility [1–4]. The higher prevalence of varicocele in men with secondary infertility than in those with primary infertility suggests that varicocele is most likely a progressive condition, rather than a static pathological condition, resulting in potential functional and structural testicular damage [5].

Several studies have explained the pathophysiology of testicular dysfunction in varicocele patients. However, the exact mechanism of infertility caused due to varicocele formation remains unclear. It is thought to be primarily related to small vessel obstruction and venous stasis in the scrotum, causing raised scrotal temperature and tissue hypoxia. This, in turn, causes germinal cell dysfunction and reduces spermatogenesis [4, 6, 7]. Other explanations for infertility in varicocele patients include endocrinological changes and the back flow of adrenal and renal metabolic products through the left internal spermatic vein [6, 8].

Another theory for the pathophysiology of infertility in varicocele patients is related to increased oxidative stress. This has been linked to sperm DNA damage, including DNA fragmentation, and correlated with the decreased capacity of spermatozoa to fertilise oocytes during normal fertilisation and assisted reproduction techniques (ARTs, in vitro fertilisation and intracytoplasmic sperm injection [ICSI]) [6, 9]. Studies have also reported a higher prevalence of DNA fragmentation in varicocele patients and a correlation between the presence of varicocele and impaired sperm DNA integrity [7, 10]. Furthermore, Zavattaro et al. reported that around 15% of varicocele patients with normal semen analysis are diagnosed as infertile [5].

The sperm DNA fragmentation index (DFI) is a potential parameter for fertility investigation. DFI reflects the sperm DNA integrity and damage [11]. Studies have suggested that the degree of DNA fragmentation can predict the outcomes of ARTs. The currently established clinical threshold is 25% DFI. A man with DFI > 25% falls into the statistical probability of encountering reproductive problems [12]. A recent study suggested that surgical repair of varicocele significantly improves sperm DNA quality [6]. A previous meta-analysis by Schauer et al. showed significant improvement in sperm parameters regardless of which surgical techniques was used (high ligation, inguinal or subinguinal techniques) [10].

Current guidelines recommend that varicoceles be treated in cases of documented infertility, palpable varicocele, normal or potentially corrected female fertility, and at least one abnormal sperm parameter [13]. High DFI, despite other normal semen parameters, has not been considered as one of the indications for varicocelectomy due to the limited number of studies available on the effects of varicocelectomy on DFI. Thus, in this study, a meta-analysis of the effects of varicocelectomy on the sperm DFI and other general parameters was performed.

## Materials and methods

### Description of condition and intervention

Studies were included if (1) the population in the studies was adult males with clinical varicoceles of grades 1–3; (2) the intervention in the studies was varicocelectomy with retroperitoneal (with high ligation), inguinal, or subinguinal surgical techniques; (3) the studies showed a comparison between pre- and post-operative sperm DFI and sperm analysis; (4) the studies used World Health Organization (WHO) criteria for sperm analysis; (5) the studies provided mean with standard deviation (SD) and 95% confidence interval (CI) values or sufficient information to calculate these values; (6) the studies were randomised controlled trials (RCTs) or cohort prospective studies; (7) the studies were published within the last 10 years; and (8) the studies used sperm chromatin structure assay (SCSA), sperm chromatin dispersion (SCD), or terminal deoxynucleotidyl transferase (TdT)-mediated-dUTP nick end labelling (TUNEL) to measure DNA fragmentation. These measurement methods are widely used in clinical settings [11, 14]. Furthermore, a study by Chohan et al. reported that SCSA, SCD, and TUNEL showed similar predictive values for DNA fragmentation [15]. A comprehensive study by Ribas-Maynou et al. reported high correlation between TUNEL and SCSA, and both assays showed high correlation with SCD, further proving that these assays showed similar predictive values for sperm DNA fragmentation [16]. To minimise bias, only studies that fit all these eligibility criteria were included in this meta-analysis.

Studies were excluded if (1) the study population was adolescent and/or presented with subclinical varicocele; (2) the studies used acridine orange staining (AOT) method to assess DFI levels (because AOT resulted in variable values of DNA fragmentation, making its use in clinical practice questionable) [15]; (3) the studies were unavailable as free full-text or in the English language; or (4) the studies were animal studies, review articles, retrospective studies, or case series.

The primary outcome of this meta-analysis was the comparison between the pre- and post-operative (varicocelectomy) sperm DFI values, while the secondary outcome was the comparison between the pre- and post-operative sperm parameters including sperm concentration, motility, and morphology.

### Database search and literature screening

A computerised literature search of the PubMed, Scopus, EBSCO, and Cochrane Library databases was performed on 23 April 2020 using the search terms ‘varicocelectomy’, ‘varicocele repair’, ‘sperm parameter’, ‘sperm analysis’, ‘semen parameter’, ‘semen analysis’, and ‘DFI’ in various combinations. We limited the search to articles published between January 2010 and May 2020. The identified articles were analysed for duplicates and screened for eligibility. The Preferred Reporting Items for Systematic Reviews and Meta-Analyses (PRISMA) guidelines were followed during this study.

### Study selection

Two reviewers (PB and RW) independently screened and appraised the articles. Any disagreement was solved by discussion until a consensus was reached. The articles were screened for relevance by reading their titles and abstracts. The articles were then assessed for eligibility according to the previously determined inclusion and exclusion criteria for this meta-analysis. The quality of each article was assessed using the Newcastle-Ottawa Scale (NOS) [17]. A total score of 6–9 on the NOS was considered high methodological quality, 4–5 was considered medium quality, and < 4 was considered low quality [18].

### Data extraction

Data of the variables, including the author, year of publication, study design, number of patients, age range, intervention, patient follow up time, DFI method, and pre- and post-operative DFI (%) values and sperm concentrations (10^6^/mL), progressive motility (%), and morphology (%), were extracted from the included articles.

### Statistical analysis

The pre- and post-operative DFI and sperm parameters were compared and analysed using the Review Manager 5.3.5 software. The results were expressed as the mean difference with 95% CI because the extracted data were of continuous variables. Heterogeneity was analysed using the Chi square and I^2^ tests. Heterogeneity was defined as *p* < 0,10 or I^2^ > 50%. Fixed-effect model was used if *p* ≥ 0,10 or I^2^ ≤ 50%. Random-effect model was used otherwise [18]. *p*-values < 0.05 were considered statistically significant.

## Results

### Literature search

**Fig. 1:**
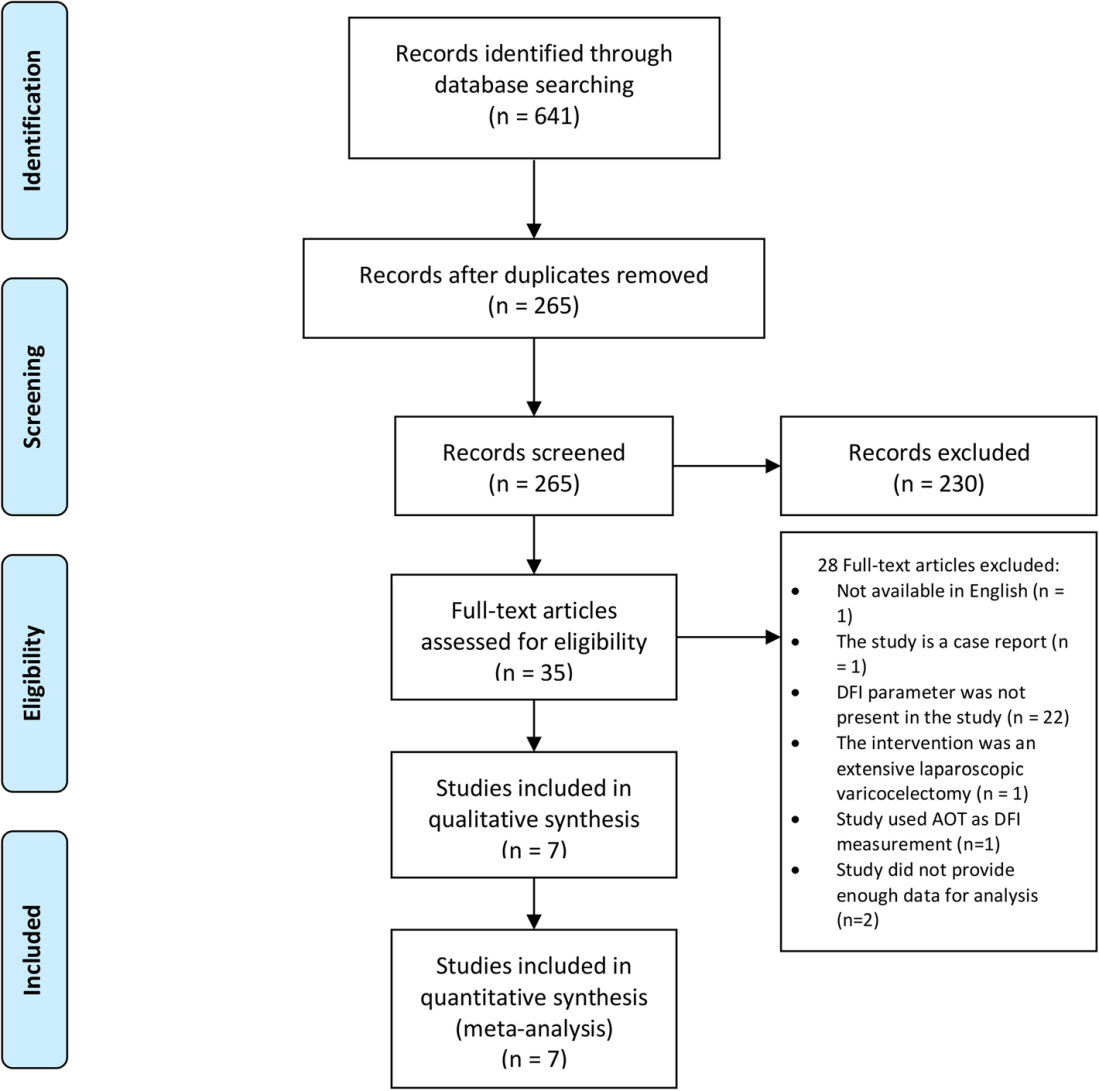
PRISMA Flow Diagram. Acridine orange staining (AOT), DNA fragmentation index (DFI), The Preferred Reporting Items for Systematic Reviews and Meta-Analyses (PRISMA)

A total of 641 articles were retrieved from the databases. After screening for duplicates, 265 articles were screened for relevance and eligibility according to the exclusion and inclusion criteria. A total of 35 articles were found to be relevant to this study. After a full-text review, 7 articles (289 patients) were included in this meta-analysis (Fig. 1).

### Study characteristics

A summary of the study characteristics is presented in Table 1. Quality assessment of the studies using the NOS is presented in Table 2 [17]. All seven studies included were prospective studies. The microsurgical varicocelectomy technique was used in the included studies, except for one study by Abdelbaki et al. [19] in which inguinal varicocelectomy with loupe was used. DFI and the other sperm parameters were examined before surgery and 3, 4, or 6 months after surgery in all the studies. No RCTs were found on searching any of the databases.

**Table 1:** Study characteristics

Reference	Design	Population (N)	Age range (years)	Age mean	Age median	Intervention	Follow up month after surgery	Outcome				
								DFI assay	Sperm concentration	Total sperm motility	Progressive sperm motility	Sperm morphology
Abdelbaki et al. [19].	Prospective	55	23–41	–	33	Inguinal varicocelectomy with loupe	3–6 months	SCSA	Yes	Yes	Yes	Yes
Alhathal et al. [20].	Prospective	29	–	–	–	Subinguinal microsurgical varicocelectomy	6 months	SCSA	Yes	No	Yes	No
Ghazi et al. [21].	Prospective	82	20–51	35.6	–	Microsurgical varicocelectomy	6 months	TUNEL	Yes	Yes	Yes	Yes
La Vignera et al. [22].	Prospective	30	20–32	26.5	–	Subinguinal microsurgical varicocelectomy	4 months	TUNEL	Yes	No	Yes	Yes
Li et al. [23].	Prospective	19	15–42	33.1	–	Subinguinal microsurgical varicocelectomy	3 months	SCSA	Yes	No	Yes	Yes
Zini et al. [24].	Prospective	25	–	–	–	Microsurgical varicocelectomy	4 months	SCSA	Yes	No	Yes	No
Smit et al. [25].	Prospective	49	34 ± 6.9	34 ± 6.9	–	Left high inguinal spermatic vein ligation and microsurgical varicocelectomy	3 months	SCSA	Yes	No	Yes	Yes

*SCSA* Sperm chromatin structure assay; TUNEL: TdT-mediated-dUTP nick end labeling

**Table 2:** Newcastle-Ottawa Scale (NOS) quality assessment of the selected articles

References	Study design	NOS	Quality
Abdelbaki et al. [19].	Prospective	8	High
Alhathal et al. [20].	Prospective	8	High
Ghazi et al. [21].	Prospective	8	High
La Vignera et al. [22].	Prospective	8	High
Li et al. [23].	Prospective	8	High
Zini et al. [24].	Prospective	8	High
Smit et al. [25].	Prospective	8	High

### DFI

**Fig. 2:**
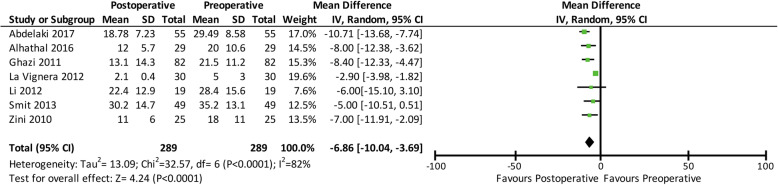
Forest plot comparing the pre- and post-operative DNA fragmentation index values. Confidence interval (CI), standard deviation (SD)

Sperm DFI was evaluated before varicocelectomy and then re-evaluated 3, 4, or 6 months after varicocelectomy in all the seven selected studies (289 participants). SCSA was used to evaluate DFI in most of the included studies, except for two studies that utilised TUNEL. After varicocelectomy, the sperm DFI decreased by 6.86% (mean difference: -6.86; 95% CI: − 10.04, − 3.69; *p* < 0.00001). A forest plot comparing the pre- and post-operative DFI values is shown in Fig. 2.

### Sperm concentration

**Fig. 3:**
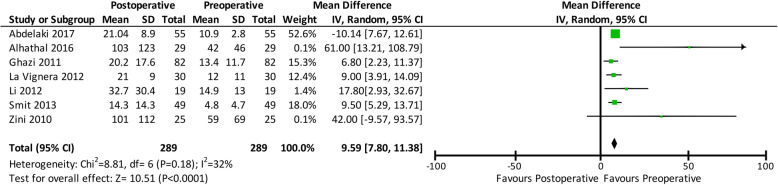
Forest plot comparing the pre- and post-operative sperm concentrations. Confidence interval (CI), standard deviation (SD)

Sperm concentration was also evaluated before and after varicocelectomy. It increased by 9.59 million per mL (mean difference: 9.59; 95% CI: 7.80, 11.38; *p* < 0.00001) after varicocelectomy. A forest plot comparing the pre- and post-operative sperm concentrations is shown in Fig. 3.

### Sperm progressive motility

**Fig. 4:**
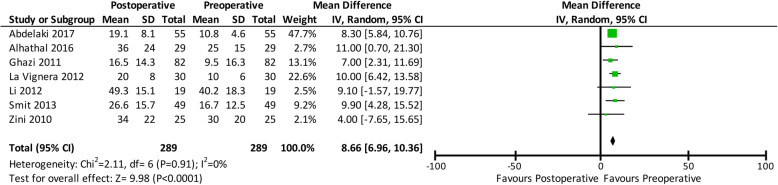
Forest plot comparing the pre- and post-operative sperm progressive motility. Confidence interval (CI), standard deviation (SD)

All studies also reported pre- and post-operative sperm progressive motility. All studies evaluated post-operative sperm progressive motility 3 months after varicocelectomy at the earliest and 6 months after varicocelectomy at the latest. The analysis (Fig. 4) revealed that progressive motility increased by 8.66% after varicocelectomy (mean difference: 8.66; 95% CI: 6.96, 10.36; p < 0.00001).

### Sperm morphology

**Fig. 5:**
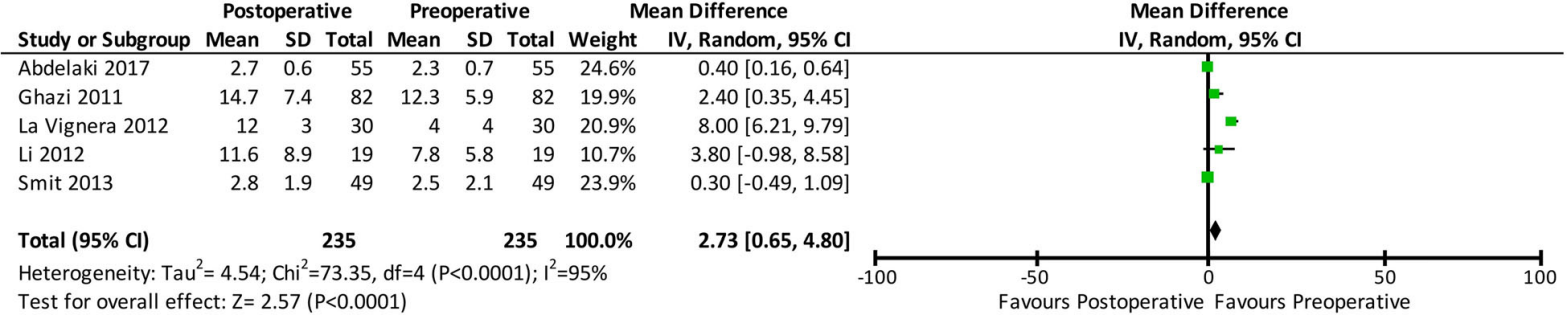
Forest plot comparing the pre- and post-operative sperm morphology. Confidence interval (CI), standard deviation (SD)

Only five out of seven studies evaluated sperm morphology before and after varicocelectomy. Sperm morphology improved by 2.73% (mean difference: 2.73; 95% CI: 0,65, 4.80; *p* = 0.01). A forest plot comparing the pre- and post-operative sperm morphology is shown in Fig. 5.

## Discussion

The guidelines dictated that the indication for varicocelectomy was infertility, as proven by abnormal sperm parameters including sperm concentration, motility, and morphology. Radiological intervention, such as sclerotherapy and embolisation, can also be used for varicocele repair. These techniques are minimally invasive; however, they result in higher recurrence rates than varicocelectomy [26]. There is also a lack of literature on the effects of sclerotherapy and embolisation on sperm DNA fragmentation.

Varicocelectomy was not recommended for infertile patients with normal semen parameters and subclinical varicocele patients [26]. According to a meta-analysis by Kroese et al. [27], the value of surgical treatment in subfertile men with subclinical varicocele and normal semen analysis is disputable. Another meta-analysis by Kim et al. [28] showed no significant difference in the pregnancy rates. Varicocele repair also showed no benefit in other instances. For example, whether men with non-obstructive azoospermia should be offered clinical varicocele treatment remains controversial [29]. However, another study reported that the sperm retrieval rate was significantly higher in men who had previously undergone varicocele repair [30].

Abnormal sperm DFI was not included as one of the indications for varicocelectomy. Furthermore, a meta-analysis by Wang et al. concluded that there was increased sperm DNA damage in varicocele patients [31]. Thus, the meta-analysis performed in the current study aimed to evaluate the effects of varicocelectomy on the sperm DFI and other parameters, including sperm concentration, motility, and morphology, and determine whether abnormal DFI levels could be considered as one of the indicators for varicocelectomy.

A total of 7 studies (289 patients) were analysed in this study. The results showed that varicocelectomy significantly reduced DNA fragmentation by 6.86% (mean difference − 6.86; 95% CI: − 10.04, − 3.69; *p* < 0.00001). The studies in this analysis used SCSA and TUNEL to assess DNA fragmentation. Using SCSA, the DNA double helix needs to be opened by denaturation process using heat or low pH to expose DNA fragments or potential DNA breaks [12]. Exposed strands then stained using acridine orange which fluoresces green when bound to native DNA and red when bound to broken DNA [32, 33]. In contrast to SCSA, TUNEL assay does not require initial denaturation step to detect DNA fragmentation [34]. In TUNEL, the addition of template-independent DNA polymerase called terminal deoxynucleotidyl transferase (TdT) on the 3′-hydroxyl (OH) free break-ends of single-strand (ss) DNA and double-stranded (ds) DNA allows measurement of DNA fragmentation [32, 35]. Each method has its own advantages and disadvantages. For example, dsDNA may have breaks with no free 3′-OH ends [36, 37]. Additionally, the reference value of DNA fragmentation for differentiating fertile and infertile men using both SCSA and TUNEL shows high variability. However, several studies have shown high correlation between SCSA and TUNEL, indicating that both assays expressed similar values of DNA fragmentation [15, 16].

Several studies not included in this analysis also showed similar results to the present study. These non- included studies were not similar to those included, because they did not fulfil the inclusion criteria. A study by Kadioglu et al., which showed a significant decrease (22.1%) in DFI after varicocelectomy, was not included in this analysis because it did not provide enough data to calculate the mean and SD. This study also showed that the higher the pre-operative DFI, the larger is the decrease in the post-operative DFI [6]. Another study by Telli et al. also revealed the decrease in DFI after varicocelectomy. However, DFI was evaluated using the AOT method [38]. AOT was reported to show higher DFI levels and variability consistently than other methods including SCSA and TUNEL [15]. These studies and our analysis showed that varicocelectomy reduced sperm DNA fragmentation. One meta-analysis by Wang et al. also proposed varicocelectomy as the potential treatment for the increase in the DNA damage in varicocele patients [31]. The effects of varicocele on the alteration in the amount of sperm DNA damage were studied in this meta-analysis. Additionally, this study aimed to determine the efficacious effects of varicocele repair on sperm DNA damage. However, some of the studies analysed in the meta-analysis by Wang et al. were retrospective studies or of unspecified study design [31]. This might have led to bias in the study results, as aforementioned in the limitations of the meta-analysis. We intended to strengthen the results of our meta-analysis by selecting only prospective studies or RCTs, if available. Although we could not find any RCT, all other studies included in the analysis were prospective studies.

An explanation for the reduction in DNA fragmentation after varicocelectomy lies in the proposed pathophysiology of DNA damage in varicocele patients. Sperm DNA fragmentation occurs during spermatogenesis and sperm maturation [11]. Hypoxia caused due to venous stasis and reflux results in an increase in reactive oxygen species (ROS). ROS directly attacks spermatozoa DNA, resulting in DNA damage and increased DNA fragmentation [39, 40]. Varicocelectomy eliminates venous stasis and reflux, thereby decreasing ROS production and thus DNA damage.

This analysis also revealed improved sperm concentration (mean difference: 9.59; 95% CI: 7.80, 11.38; *p* < 0.00001), progressive motility (mean difference: 8.66; 95% CI: 6.96, 10.36; p < 0.00001), and morphology (mean difference: 2.73; 95% CI: 0,65, 4.80; *p* = 0.01) after varicocelectomy. We also evaluated the effects of varicocelectomy on these sperm parameters, in addition to sperm DFI, to strengthen the evidence for the correlation between varicocelectomy and sperm parameters. A previous meta-analysis reported the positive effects of three different varicocelectomy surgical techniques on sperm parameters, and the results of this study were in conjunction with those of our meta-analysis [10].

Our analysis showed a potential negative correlation between DFI and the other sperm parameters before and after varicocelectomy. Several studies have reported the correlation between DFI and other sperm parameters. Kadioglu et al. reported that higher pre-operative DFI was associated with significant negative correlation between DFI and sperm motility (r = − 0.42, *p* < 0.01) [6]. Telli et al. also reported a negative correlation between DFI and sperm motility (r = − 0.267, *p* = 0.043) [38]. Yang et al. reported a negative correlation between DFI and sperm motility, concentration, and morphology (r = − 0.307, − 0.552, and − 0.620, respectively; all p < 0.01) [11]. This study also revealed a positive correlation between DFI and age, suggesting that DFI increased as the patient aged. Furthermore, a study by Smit et al. reported that low DFI values were associated with higher rates of pregnancy, both spontaneous and though ARTs [25]. All these evidences suggested that DFI might be as important as other sperm parameters for evaluating male fertility and fertility after varicocelectomy for a successful pregnancy.

The limitations of this meta-analysis include the heterogeneity of the included studies in terms of sample size and methods of intervention and evaluation of the outcomes. All included studies were prospective studies. Even though this was ideal, inclusion of RCTs could further strengthen the study analysis. Unfortunately, no RCT was found during the literature search of the databases. However, one preliminary RCT was found on manual searching, but this RCT included only five patients who underwent varicocelectomy and showed no before-after DFI data or *p*-value data, making the study undetermined for significance [41]. This difficulty in finding RCTs could be due to the ethical problems in varicocelectomy being performed in only one group of clinical varicocele patients and not in the other group, despite their need to receive varicocelectomy as a treatment for infertility.

## Conclusions

Varicocelectomy reduces DNA fragmentation and improves sperm concentration, progressive motility, and morphology. In the current guidelines, alterations in only sperm parameters including sperm motility and morphology are considered as indications for varicocelectomy. Thus, we performed a meta-analysis that showed that abnormal DFI measurement should also be considered as an indication for varicocelectomy.

## Data Availability

Not applicable.

## Abbreviations

AOT: Acridine orange staining
ART: Assisted reproduction technique
CI: Confidence interval
DFI: DNA fragmentation index
DNA: Deoxyribonucleic acid
ds DNA: Double-stranded DNA
ICSI: Intracytoplasmic sperm injection
NOS: Newcastle-Ottawa Scale
PRISMA: Preferred Reporting Items for Systematic Reviews and Meta-Analyses
RCT: Randomised controlled trial
ROS: Reactive oxygen species
SCD: Sperm chromatin dispersion
SCSA: Sperm chromatin structure assay
SD: Standard deviation
ssDNA: Single-strand DNA
TUNEL: TdT-mediated-dUTP nick end labelling
WHO: World Health Organization
